# Development of a New Internally Controlled One-Step Real-Time RT-PCR for the Molecular Detection of Enterovirus A71 in Africa and Madagascar

**DOI:** 10.3389/fmicb.2020.01907

**Published:** 2020-08-14

**Authors:** Romain Volle, Marie-Line Joffret, Kader Ndiaye, Maria Dolores Fernandez-Garcia, Richter Razafindratsimandresy, Jean-Michel Heraud, Dorra Rezig, Serge Alain Sadeuh-Mba, Leila Boulahbal-Anes, Mohamed Seghier, Jagadish M. Deshpandeh, Maël Bessaud, Francis Delpeyroux

**Affiliations:** ^1^Institut Pasteur, Unité de Biologie des Virus Entériques, Paris, France; ^2^INSERM U994, Institut National de Santé et de La Recherche Médicale, Paris, France; ^3^Institut Pasteur, Viral Populations and Pathogenesis, Paris, France; ^4^Institut Pasteur de Dakar, Dakar, Senegal; ^5^Institut Pasteur de Madagascar, Antananarivo, Madagascar; ^6^Institut Pasteur de Tunis, Tunis, Tunisia; ^7^Centre Pasteur du Cameroun, Yaoundé, Cameroon; ^8^Institut Pasteur d’Algérie, Algiers, Algeria; ^9^National Institute of Virology, Indian Council of Medical Research (ICMR), Mumbai, India

**Keywords:** enterovirus 71, EV-A71, HFMD, new genogroups, molecular detection

## Abstract

Enterovirus A71 (EV-A71) is a leading cause of hand-foot-and-mouth disease (HFMD) and can be associated with severe neurological complications. EV-A71 strains can be classified into seven genogroups, A-H, on the basis of the VP1 capsid protein gene sequence. Genogroup A includes the prototype strain; genogroups B and C are responsible of major outbreaks worldwide, but little is known about the others, particularly genogroups E and F, which have been recently identified in Africa and Madagascar, respectively. The circulation of EV-A71 in the African region is poorly known and probably underestimated. A rapid and specific assay for detecting all genogroups of EV-A71 is required. In this study, we developed a real-time RT-PCR assay with a competitive internal control (IC). The primers and *Taq*Man probe specifically target the genomic region encoding the VP1 capsid protein. Diverse EV-A71 RNAs were successfully amplified from the genogroups A, B, C, D, E, and F, with similar sensitivity and robust reproducibility. Neither cross reaction with other EVs nor major interference with the competitive IC was detected. Experimentally spiked stool and plasma specimens provided consistent and reproducible results, and validated the usefulness of the IC for demonstrating the presence of PCR inhibitors in samples. The analysis in an African laboratories network of 1889 untyped enterovirus isolates detected 15 EV-A71 of different genogroups. This specific real-time RT-PCR assay provides a robust and sensitive method for the detection of EV-A71 in biological specimens and for the epidemiological monitoring of EV-A71 including its recently discovered genogroups.

## Introduction

Human enterovirus A71 (EV-A71) is a member of species A of the genus *Enterovirus* (*Picornaviridae*). Enteroviruses form a large group of human pathogens, with hundreds of different types, the best known being the three types of poliovirus responsible for poliomyelitis (Pallansch and Roos, 2007). EV-A71 is one of the leading causes of childhood hand-foot-and-mouth disease (HFMD), together with coxsackievirus A6, A10 and A16. Unlike other HFMD-associated enteroviruses, EV-A71 frequently elicits serious neurological complications, including meningitis, encephalitis, and polio-like flaccid paralysis (McMinn, 2002; Ooi et al., 2010; Solomon et al., 2010). With major outbreaks in the Asia-Pacific region during the 1990s and its implication in fatal cardiopulmonary edema (Biswas, 2012; Horwood et al., 2016), EV-A71 has emerged as a serious public health concern for local authorities. In 2011, the World Health Organization (WHO) published its Guidelines for Clinical Management of HFMD (World Health Organization [WHO], 2011). Asian-Pacific countries have developed surveillance systems, and EV-A71 circulation is also being followed by surveillance laboratories in some European countries (van der Sanden et al., 2009; Mirand et al., 2010; Fischer et al., 2014).

Molecular epidemiology studies based on the genomic sequence 1D encoding the VP1 protein (1D^VP1^) led to the classification of EV-A71 strains into genogroups A to H (Bessaud et al., 2014; Majumdar et al., 2018). Genogroups B and C are the best known, because they have been reported worldwide in most outbreaks and individual cases for decades. These two canonical genogroups were further subdivided into subgenogroups B0 to B5 and C1 to C5. The other EV-A71 genogroups are less well characterized. Genogroup A includes the prototype strain (BrCr) isolated in 1969. Genogroups D and G were reported in India (Saxena et al., 2015). Genogroup E was reported in Central African Republic (Bessaud et al., 2012), Cameroon (Sadeuh-Mba et al., 2013), and Senegal (Fernandez-Garcia et al., 2016). Genogroup F was specifically reported in Madagascar (Bessaud et al., 2014). No outbreaks due to EV-A71 have yet been reported in Africa and Madagascar, but the circulation of EV-A71 and its impact on human health may be underestimated. Elucidation of EV-A71 evolutionary connections between African strains and those circulating in the rest of the world would improve the global EV-A71 surveillance (McWilliam Leitch et al., 2012; Hassel et al., 2015).

Over the last decade, highly sensitive and specific real-time RT-PCR tests have been developed to detect enteroviruses in various biological specimens (stools, throat swabs and cerebrospinal fluid) and in cell culture supernatants. Real-Time RT-PCR is now the gold standard for EV-A71 detection during HFMD outbreaks in the Asia-Pacific region (Xiao et al., 2009; Hwang et al., 2013; Zhang et al., 2014; Thanh et al., 2015). However, previous real-time RT-PCR assays use primers and probe sets mostly designed from the sequences of EV-A71 genogroups B and C.

We report here the development, validation and application of a new pan-EV-A71 one-step real-time RT-PCR assay based on primers and *Taq*Man probes capable of detecting most of EV-A71 strains belonging to both the canonical B and C and the recently described D, E and F genogroups. To detect RT-PCR inhibitors, an internal control (IC) was included in the assay. First data following its robust multicenter implementation within an African laboratory network led to the finding of EV-A71 isolates and indicated that this assay will allow an efficient EV-A71 surveillance in this continent.

## Materials and Methods

### Virus Isolates

The EV-A71 prototype strain genogroup A (BrCr), and isolates from subgenogroup C4 (C4-SEP06) (Horwood et al., 2016), subgenogroup C1 (C1-CAE041) (Sadeuh-Mba et al., 2013), subgenogroup B2 (B2-CHE516_DEU87; Robert Koch Institute), genogroup E (E-CAE146 and E-CAF008) (Bessaud et al., 2012; Sadeuh-Mba et al., 2013), and genogroup F (F-MAD72341 and F-MAD3126) (Bessaud et al., 2014) were used as reference strains to assess the sensitivity and reproducibility of the assay for detecting the various EV-A71 genogroups. Clinical samples known to contain EV-A71 strains belonging to genogroups D and G were also tested in collaboration with the Enterovirus Research Centre of Mumbai, India (Saxena et al., 2015). Reference strains of coxsackieviruses A2, A3, A4, A6, A7, A8, A10, A12, A14, A16, B1 to B6; echoviruses 4, 6, 11, 13, 25, and 30 were from the European Virus Archive EVAg^1^. Poliovirus-1 (attenuated Sabin strain – WHO) and EV-D68 (EVAg) were used as negative controls, to assess the specificity of the assay to EV-A71 strains. Viruses were propagated in Vero/E6 or RD cells. Virus infectivity was assayed in microplates, by calculating the 50% tissue culture infective dose (TCID_50_) according to the Spearman–Kärber method (Ramakrishnan, 2016).

### Biological Specimens

We obtained 20 biological specimens from ICAReB Biobank, Institut Pasteur, Paris (**C**linical **I**nvestigation and **A**ccess to **Re**search **Bi**o-resources). All specimens were obtained in accordance with national and international ethics requirements, with the informed consent of adults healthy volunteers from the ICAReB’s Diagmicoll cohort, whose protocol was approved by the Committee of Protection of Persons Ile de France-1 (2008, April 30) (Esterre et al., 2020). The studied samples consisted of 10 stool specimens and 10 heparin or EDTA plasma samples.

### Design of Primers and Probes for the Real-Time RT-PCR

Highly conserved sequences from the 1D^VP1^ gene of EV-A71 were used as target sites for the design of specific primers and probes. Nucleotide sequences were retrieved from the GenBank database with the following combination of keywords: “enterovirus 71” AND “1D”. Editing was performed manually and exploratory alignments were generated with BioEdit software. Sequences that were wrongly annotated or incomplete were not considered. Overall 2945 eligible sequences (from 2945 isolates) were finally aligned with the prototype 1D^VP1^ gene of the genogroup A EV-A71/BrCr strain (GenBank accession no: AB204852.1). Conserved sequences regions within the alignment were then identified, with the following parameters: no gaps allowed, a minimum length of 15 nucleotides, a maximum average and per position entropy of 0.2. The generated consensus sequences for each conserved region were then compared with the 1D^VP1^ gene of all EV prototype strains, and those that seemed to be most specific to EV-A71 were selected, to design the degenerate sequences of the primers and the *Taq*Man probe. The EV-A71 *Taq*Man and the IC probes were labeled at their 5′ ends with the fluorescent dyes 6-carboxy-fluorescein (6FAM) and hexachloro-6-carboxy-fluorescein (HEX), respectively, and at their 3′ ends with black hole quencher 1 (BHQ1). Oligonucleotides were manufactured by Sigma-Aldrich (St Quentin Fallavier, France) and Eurofins-Genomics (Ebersberg, Germany) based on their specific sequences provided in Table 1.

**TABLE 1:** Nucleotidic sequences of primers, probes, and of the internal control (IC).

**Primers and probes**		**Sequence (5**′**–3**′**)^a^**	**Nucleotide position^b^**	**References**
**EV-A71 real-time RT-PCR**				
	EV-A71 Forward primer	ATGATGGG**H**AC**N**TTCTC	3,123-3,139	/
	EV-A71 Reverse primer	GA**N**TT**N**CC**D**GC**R**TA**V**TTTGG	3,267-3,286	/
	TaqMan EV-A71 Probe	(6FAM)-AAGCA**Y**GTCAG**R**GC**N**TGG**R**TACC-(BHQ1)	3,204-3,226	/
	TaqMan IC Probe	(HEX)-CCTGTGGAACACCTACATCTGT-(BHQ1)	/	/
	Internal Control (IC)^c^	CGTAAAGTCTTAATACGACTCACTATAGGGAGTCTGCCACCATGGATGATGG GCACGTTCTCAAGTCAGCGCCCTGCACCATTATGTTCCGGATCTGCATCGC AGGATGCTGCTGGCTACCCTGTGGAACACCTACATCTGTATTAACGAAGCG CTGGCATAGACCCTCAGTGATTTTTCTCTCCAAATTACGCTGGCAATTCCAT CCATACCGCTAAGCTAGTTATTGCTCAGCGGCGTAAAGTCT	/	/
**EV gene sequencing**				
	AN224	GC**I**ATG**Y**T**I**GG**I**AC**I**CA**YR**T	2,157-2,176	Nix et al., 2006
	AN222	C**I**CC**I**GG**I**GG**I**A**YRW**ACAT	2,888-2,912	
	C004-F	TTAAAACAGC**YYKD**GGGTTG	1-20	Bessaud et al., 2016
	EV-CRE-R	CGG**BR**TTT**GSW**CTTGAACTG	4,413-4,432	Joffret et al., 2018

*^*a*^Bold letters indicate degenerated nucleotidic bases. ^*b*^EV-A71/BrCr complete genome; Genbank Accession nb: AB204852.1. ^*c*^Underlined letters indicate the hybridization site of the EV-A71 forward primer, TaqMan IC Probe, and reverse primer, respectively.*

### Preparation of the Internal Control RNA

The design and preparation of the competitive IC was based on methods used in previous studies (Volle et al., 2012). A chimeric sequence based on pBR322 was designed to contain the EV-A71 primer-binding sites and the specific pBR322 IC probe-binding site. The EV-A71 primer-binding sites inserted into the IC could not include degenerate bases, which were, thus, randomly replaced with regular bases, maintaining a G/C content of 51% (see the IC sequence, Table 1). The 5′-end includes an *Eco*RI restriction site followed by the T7 RNA polymerase promotor and the binding site for the EV-A71 forward primer. The 3′-end includes the binding site for the EV-A71 reverse primer, followed by a T7 terminator and a *Hin*dIII restriction site. The 259 nucleotide-long IC DNA fragment was synthesized *in vitro*, inserted into the pEX-A2 vector and checked by sequencing (performed by Eurofins Genomics, Ebensberg, Germany). The recombinant plasmid containing the IC DNA was linearized with *Bam*HI and transcribed *in vitro* with the RiboMax^TM^ Large-Scale RNA Production system-T7 (Promega, Madison, WI, United States). The DNA template was then removed by DNase treatment following the RiboMax^TM^ Large-Scale RNA Production system-T7 protocol and RNA was quantified with a NanoDrop spectrophotometer (Thermo Fisher Scientific, France).

### Preparation of the Positive Control EV-A71 RNA

The 1D^VP1^ gene of the EV-A71 subgenogroup C4 SEP6 strain was inserted into pCRII^®^-TOPO^®^Vector according to the manufacturer’s instructions (TOPO TA Cloning Kit Dual Promoter, Invitrogen, Thermo Fisher Scientific, France). The recombinant plasmid was then linearized with *Hin*dIII and the cloned 1D^VP1^ gene was transcribed *in vitro* with the RiboMax^TM^ Large-Scale RNA Production system-T7 (Promega, Madison, WI, United States). The DNA template was then removed by DNase treatment following the RiboMax^TM^ Large-Scale RNA Production system-T7 protocol and RNA was quantified with a NanoDrop spectrophotometer (Thermo Fisher Scientific, France).

### Biological Samples Processing and RNA Extraction

Stool samples were resuspended as a 20% (g/ml) suspension in PBS. Aliquots (200 μl) of this suspension were experimentally spiked with EV-A71 C4-SEP06, to obtain a stool suspension with 10 TCID_50_/ml of virus, as shown by titration on Vero cells. The spiked stool suspensions were treated with 20% (v/v) chloroform for 10 min, with vigorous vortexing, and clarified by centrifugation at 1,500 × *g* for 10 min, as recommended by the WHO HFMD guidelines (World Health Organization [WHO], 2011). Aliquots (200 μl) of plasma were similarly spiked with EV-A71 C4-SEP06 (10 TCID_50_/ml final).

Viral RNAs from infected cell suspensions, clarified stool supernatants or plasma were extracted with the Virus RNA Min Elute extraction kit (Roche, Mannheim, Germany), according to the manufacturer’s instructions. The IC RNA (10^5^ copies per sample) was added to the extraction tube before the lysis step. The inhibitory clean-up step was performed twice, to extract viral RNA from spiked plasma. The purified RNA was eluted in 50 μl of elution buffer and immediately analyzed or stored at −80°C until use.

### Real-Time RT-PCR Conditions

Real-time RT-PCR was carried out with the Superscript^TM^ III Platinum^TM^ One-step Quantitative Kit (Invitrogen, Thermo Fisher Scientific, France). We added 5 μl RNA to 15 μl of reaction mixture containing reaction mix buffer (1×), each primer and probe at a concentration of 0.5 μM, and 0.4 μl SuperScript^TM^ III RT/Platinum^TM^ *Taq*Mix. A one-step real-time RT-PCR was performed with the 7500 real-time PCR system (ABI), in 96-well plates, under the following conditions: reverse transcription at 50°C for 30 min, *Taq* DNA polymerase activation at 95°C for 2 min, and then 45 cycles of amplification consisting of DNA denaturation for 15 s at 95°C, primer and probe annealing at 50°C for 30 s, and extension at 72°C for 1 min. Fluorescence data were collected at the end of each cycle. The EV-A71 and the IC RT-PCR products have lengths of 139 and 158 bp, respectively.

### Surveillance of the Circulation and Diversity of EV-A71 in Africa

This survey has been conducted in collaboration with the Instituts Pasteur of Alger, Tunis, Dakar and Antananarivo, and the Centre Pasteur du Cameroun at Yaoundé. Non-poliovirus isolates or in some cases clinical samples from poliovirus surveillance collected from 2000 to 2016 were retrospectively analyzed for EV-A71 with the real-time RT-PCR as described. Sequencing of the 1D^VP1^ genes was performed using the methods described in Nix et al. (2006) and Oberste et al. (2000). In some cases high-throughput sequencing was used as described using the primers C004 (Bessaud et al., 2016) and EV-CRE-R (Joffret et al., 2018) (see Table 1). The EV-A71 phylogram based on the gene sequences of 1D^VP1^ (891 nucleotides) was reconstructed with Neighbor Joining (NJ) method using MEGA6 software (Tamura et al., 2013) with the Kimura 2-parameter evolutionary model and 1,000 bootstrap replicates for nodes consistency estimation.

### Sequence Data Accession Number

Sequencing data are publicly available from the European Nucleotides Archives browser and assigned to the following accession number (LR798434-LR798440).

## Results

### Development and Detection Limit of the Monoplex Real-Time RT-PCR for EV-A71

**FIGURE 1:**
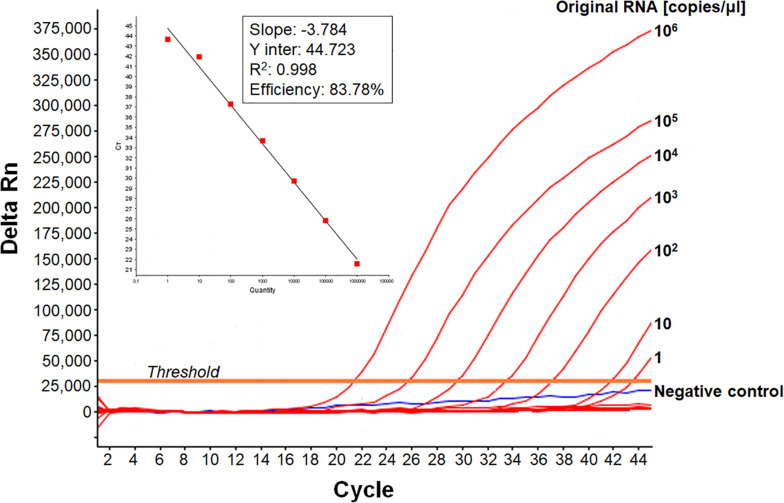
Sensitivity of the real-time RT-PCR for detecting EV-A71 C4-SEP06 RNAs. The cloned 1D^VP1^ gene of the C4-SEP06 strain was transcribed *in vitro* and then subjected to serial 10-fold dilutions from 10^6^ to 0.1 copies/μl in nuclease-free water. The linear regression line is shown with its associated correlation coefficient (*R*^2^ value) and its amplification efficiency (shown as a percentage), as given by ABI 7500 software. The negative control (Nuclease-free water) is shown in blue.

**FIGURE 2:**
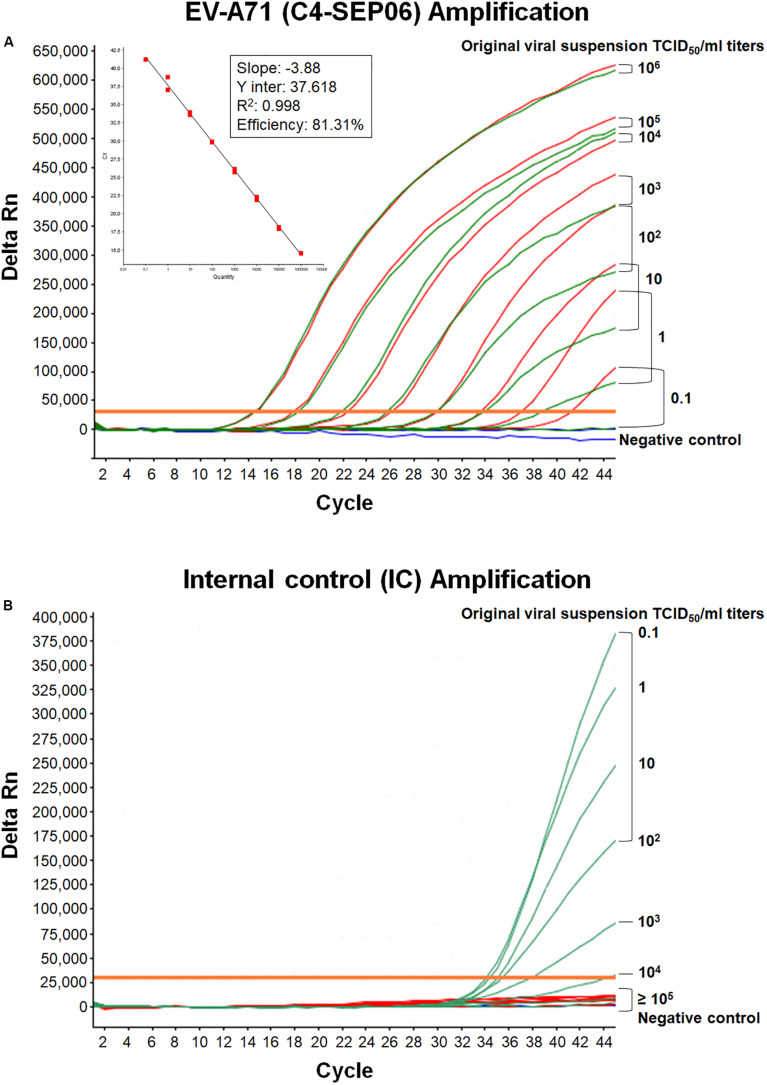
Mutual interference between EV-A71 and IC. **(A)** RNA genomes were extracted from 10-fold serially diluted C4-SEP06 viral suspensions (from 10^6^ to 0.1 TCID_50_/ml in DMEM medium) with (green curves) or without (red curves) a fixed amount of IC RNA (10^5^ RNA copies). They were then subjected to amplification in the same duplex real-time RT-PCR. The linear regression line is shown with its associated correlation coefficient (*R*^2^ value) and the amplification efficiency (shown as a percentage), as given by the ABI 7500 software. The negative control (DMEM medium) is shown in blue. **(B)** The IC RNA curves corresponding to the green EV-A71 RNA curves in panel **(A)**.

We developed a real-time RT-PCR assay for detecting various EV-A71 genogroups, by aligning most of the EV-A71 sequences available from databases and designing degenerate primers and one probe targeting the 1D^VP1^ genomic region encoding the VP1 capsid protein (Table 1). The assay was based on the amplification of a 139-nucleotide cDNA fragment detected by hybridization with a specific *Taq*Man probe. The limit of detection (LOD) was evaluated by serially diluting EV-A71 suspensions and/or *in vitro*-transcribed 1D^VP1^ RNA solutions. The LOD of the assay was first evaluated with an Asian subgenogroup C4 isolate (C4-SEP06), which was used as the standard positive control. The LOD with the *in vitro*-transcribed RNAs was estimated at 10 RNA copies/μl (Figure 1), with a mean inter-assay threshold cycle (four independent replicates) of 42.3 and a standard deviation of 0.4 (42.3 ± 0.4). The linear regression lines generated by plotting Ct values against RNA copies/μl gave coefficients of correlation (*R*^2^) ranging from 0.985 to 0.998 and an amplification efficiency of 74.0–83.8%. The LOD for C4-SEP06 viral suspensions was 0.1 TCID_50_/ml (Figure 2A, see red lines), with a mean inter-assay threshold cycle of 40.2 ± 0.7 (Table 2). The linear regression lines generated by plotting Ct values against TCID_50_/ml gave coefficients of correlation, *R*^2^, ranging from 0.993 to 0.999 and an amplification efficiency of 83.2–87.7% (Table 2).

**TABLE 2:** Reproducibility of the EV-A71 real-time RT-PCR with or without internal control.

**EV-A71 C4-SEP06 (TCID50/ml)**	**EV-A71 template^a^**																	
	**Without IC**						**With IC**						**IC template^b^**					
	**Replicate Ct value**				**Ct mean (±SD)**	**%CV^c^**	**Replicate Ct value**				**Ct mean (±SD)**	**%CV^c^**	**Replicate Ct value**				**Ct mean (±SD)**	**%CV^c^**
	**1**	**2**	**3**	**4**			**1**	**2**	**3**	**4**			**1**	**2**	**3**	**4**		
10^6^	14.6	14.7	15.1	14.2	14.7 (±0.4)	2.7	14.5	13.9	14.4	15.2	14.5 (±0.5)	3.4	NA	NA	NA	NA	–	–
10^5^	17.9	18.3	17.9	18.4	18.1 (±0.3)	1.7	18.1	18.2	18.1	19.2	18.4 (±0.5)	2.7	NA	NA	NA	NA	–	–
10^4^	22.3	22.3	22.2	22.9	22.4 (±0.3)	1.3	21.8	21.8	22.2	22.9	22.2 (±0.5)	2.3	NA	44.1	NA	NA	–	–
10^3^	26.1	26.0	26.1	27.1	26.3 (±0.5)	1.9	25.7	26.0	26.4	26.8	26.2 (±0.5)	1.9	38.9	37.9	39.7	38.7	38.8 (±0.7)	1.9
10^2^	29.9	29.8	29.5	29.3	29.6 (±0.3)	1.0	29.8	29.8	30.0	30.7	30.1 (±0.4)	1.3	36.1	35.4	35.7	35.9	35.8 (±0.3)	0.8
10	33.6	33.6	33.7	34.2	33.8 (±0.3)	0.9	33.9	33.7	34.6	34.7	34.2 (±0.5)	1.5	34.8	35.0	35.9	34.9	35.2 (±0.5)	1.4
1	37.0	37.0	38.0	36.9	37.2 (±0.5)	1.3	38.8	38.8	37.7	40.8	39.0 (±1.3)	3.3	35.4	35.0	35.7	34.9	35.3 (±0.4)	1.0
0.1	41.2	39.8	39.7	39.9	40.2 (±0.7)	1.7	NA	NA	NA	NA			34.2	34.4	34.7	35.1	34.6 (±0.4)	1.1
(*R*^2^)^d^	0.999	0.998	0.995	0.993			0.998	0.998	0.996	0.994								
Efficiency^e^	83.2	87.7	86.5	87.2			77.6	76.4	74.6	74.7								

*Ct, threshold cycle; SD, standard deviation; CV, coefficient of variation; NA, not amplified. ^*a*^EV-A71 suspension were complemented or not with a fix amount of Internal Control (IC) and RNAs were extracted and tested. ^*b*^Ct value given by the fix amount of the IC template in the presence of the corresponding EV-A71 concentrations, as mentioned in the first column. ^*c*^%CV = (SD / Ct mean) × 100. ^*d*^Linear relationship, coefficient of correlation of the 10-fold serially diluted C4-SEP06 viral suspension. As given by ABI 7500 software. ^*e*^Amplification efficiency of the 10-fold serially diluted C4-SEP06 viral suspension. As given by ABI 7500 software.*

We also determined the LOD of the assay for the different EV-A71 (sub)genogroups: A, C1, B2, E, and F. LOD was estimated at 1 TCID_50_/ml for the A-BrCr, B2-CHE516, C1-CAE041, E-CAE146 and F-MAD3126 suspensions, and at 10 TCID_50_/ml for the E-CAF008 and F-MAD72341 suspensions (see next paragraph for other details and figures).

In summary, the assay thus displayed reliable sensitivity for various EV-A71 isolates from different genogroups, with a consistent LOD.

### Simultaneous Amplifications of EV-A71 and Internal Control Templates by Duplex Real-Time RT-PCR

To monitor the functionality of the real-time RT-PCR assay and to check for the absence of inhibitors in tested samples, a synthetic exogenous competitive RNA IC was introduced. The assay was developed to amplify the EV-A71 and IC templates simultaneously. The corresponding templates compete for primer binding, but can be differentiated through their own fluorescent *Taq*Man probes. The optimal IC concentration was determined as the smallest amount of IC that could be reproducibly amplified without affecting the amplification of the EV-A71 template in the duplex real-time RT-PCR assay. The LOD of the IC was determined by using 10-fold serial dilutions (from 10^6^ to 0.1 copies/μl) of IC RNA in two independent monoplex RT-PCR assays and was estimated at 10^2^ RNA copies/μl (5 × 10^3^ RNA copies per sample), with a late threshold cycle at 43.5 and low-intensity fluorescent signal (not shown). However, the frequent failure of IC amplification due to competition with the EV-A71 template in the duplex RT-PCR assay led us to increase the IC concentration to 2 × 10^3^ copies/μl per RT-PCR run (10^5^ RNA copies per sample).

Mutual interference between the EV-A71 and IC templates during amplification was assessed in four independent assays, by determining Ct values and amplification efficiency for serial dilutions of the EV-A71 C4-SEP06 suspension (from 10^6^ to 0.1 TCID50/ml) with or without prior addition of the predetermined fixed amount (10^5^ copies) of IC RNA (Figures 2A,B). Results indicated a decrease in sensitivity of 0.1–1.0 TCID_50_/ml for C4-SEP06 in the presence of IC, with a mean inter-assay threshold cycle of 39.0 ± 1.3. *R*^2^ values ranged from 0.994 to 0.998 and amplification efficiency ranged from 74.6 to 77.6% (Table 2). The IC template was efficiently amplified in assays, from the lowest concentrations of C4-SEP06, 0.1 TCID_50_/ml, to 10^2^ TCID_50_/ml, with a global reproducible mean Ct value of 35.2 ± 0.6 (Table 2). IC amplification was completely inhibited by the presence of larger amounts of EV-A71 template (Figure 2B and Table 2).

**FIGURE 3:**
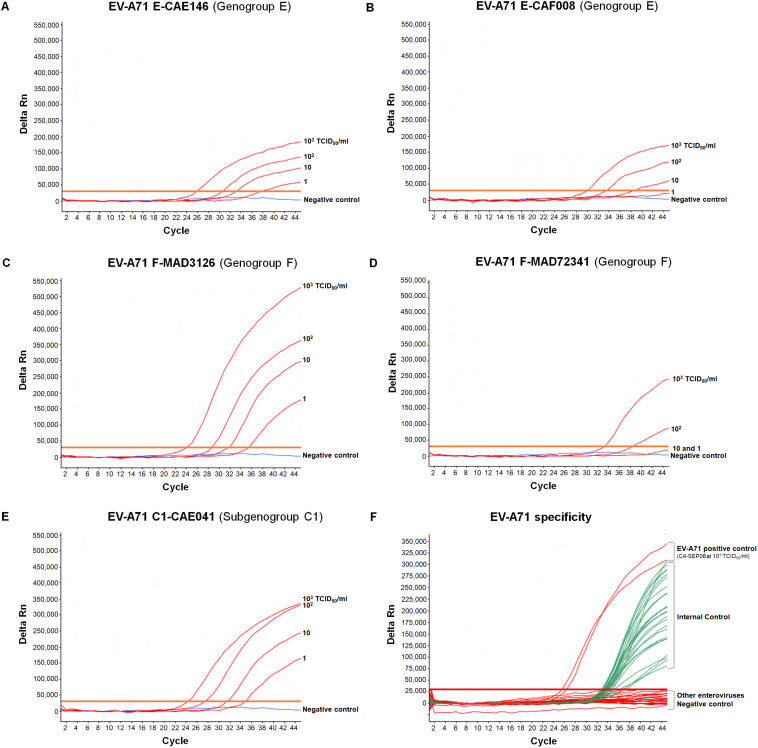
Specificity and sensitivity of the real-time RT-PCR assay for different EV-A71 genogroups. **(A**–**E)** RNA genomes were extracted from 10-fold serial dilutions of EV-A71 suspensions (from 10^3^ to 1 TCID_50_/ml in DMEM medium) in the presence of a fixed amount of IC (10^5^ RNA copies), and tested. EV-A71 template (red), negative control (DMEM medium, blue), only EV-A71 amplification is represented. **(F)** RNA was extracted from undiluted suspensions of reference strains of other enteroviruses in the presence of a fixed amount of IC (10^5^ RNA copies) and tested. Coxsackieviruses A2, A3, A4, A6, A7, A8, A10, A12, A14, A16, coxsackieviruses B1–B6, echoviruses 4, 6, 11, 13, 25, and 30, poliovirus-1 (Sabin strain), EV-D68 and EV-A71 were tested. Enterovirus RNA curves are shown in red, IC curves are shown in green, and the negative control is shown in blue. For clarity, the EV-A71 and IC amplifications are represented on the same graph.

Interference between IC and EV-A71 templates was also evaluated with the African EV-A71 isolates. Identical LODs of 1 TCID_50_/ml for E-CAE146, F-MAD3126, and C1-CAE041 and of 10 TCID_50_/ml for E-CAF008 were obtained in the presence or absence of IC (Figures 3A–C,E). However, the LOD for F-MAD72341 increased from 10 to 10^2^ TCID_50_/ml when the IC was co-amplified (Figure 3D). Higher LODs (10 TCID_50_/ml) were also obtained in the presence of IC for the BrCr and the B2-CHE516 strains.

The addition of appropriate amounts of IC RNA to the samples thus had little effect on the overall sensitivity of the assay for detecting EV-A71 sequences from various genogroups.

### Robust Inter- and Intra-Assay Reproducibility of the Real-Time RT-PCR

The reproducibility of the assay between and within runs was evaluated in the presence and absence of the IC RNA, by calculating the concentration-dependent coefficients of variation (%CV) for each dilution point of the C4-SEP06 positive control, by dividing the standard deviation of the Ct values by the mean Ct value.

The inter-assay coefficient of variation for the four independent assays was below 3.4% for the C4-SEP06 viral suspension (see Table 2). Intra-assay coefficients of variation were below 1.6% for quadruplicates in the same run, for some dilutions of the viral suspension.

Overall, inter- and intra-assay reproducibility of the data was good, either in the presence or absence of the IC RNA.

### Specificity of the Real-Time RT-PCR Assay for EV-A71

We evaluated the strict specificity of the primer and probe sets and, thus, of the RT-PCR assay, for the EV-A71 serotype, by testing suspensions of different enterovirus reference strains belonging to the four species infecting humans (EV-A to D; see the comprehensive list in the legend to Figure 3). All enteroviral suspensions resulted from complete cell culture lysis and were tested undiluted.

No amplification by real-time RT-PCR was recorded for all non-EV-A71 enterovirus strains. The IC was efficiently amplified in all tests, with consistent Ct values (Figure 3F). The assay was, therefore, specific for EV-A71 of genogroups A, B, E, and F (see above) as well as for the genogroup D that was tested in collaboration with the Enterovirus Research Centre of Mumbai (India). Unfortunately, the two available samples known to contain EV-A71 of genogroup G failed to give a positive result.

### Detection of EV-A71 in Stool and Plasma Biological Specimens

We evaluated the usefulness of the RT-PCR assay for the direct detection of EV-A71 in biological specimens. Aliquots of suspensions from 10 stool specimens and 10 plasma samples were used. Aliquots were experimentally spiked with the EV-A71 C4-SEP06 viral suspension (10 and 10^3^ TCID_50_/ml final for stool and plasma specimen, respectively), and then subjected to RNA extraction with prior addition of IC (10^5^ RNA copies per sample). Non-spiked aliquots were used as negative controls.

**FIGURE 4:**
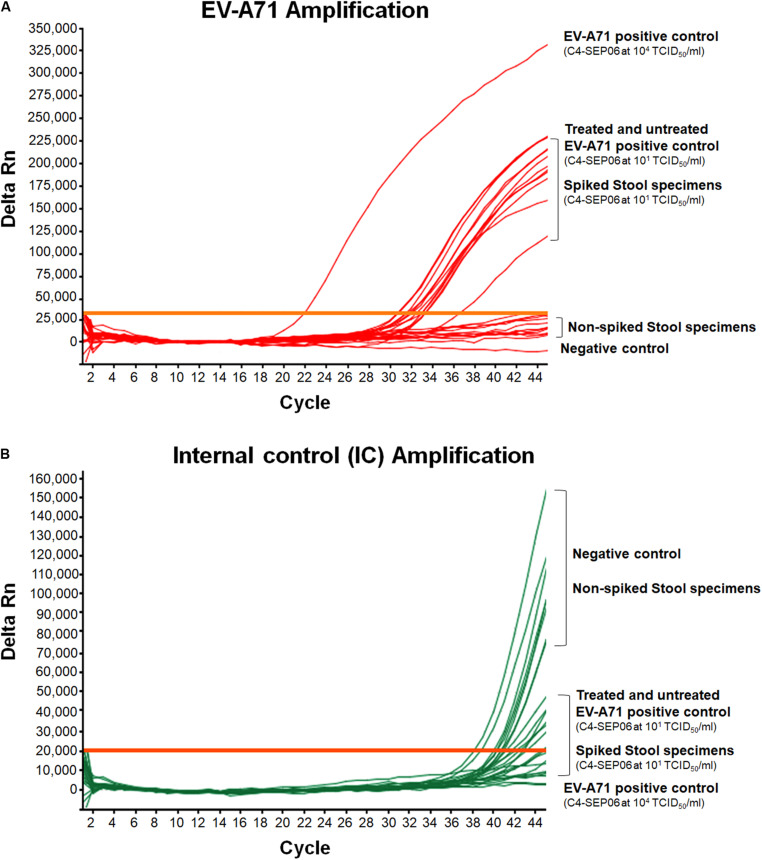
Application of the real-time RT-PCR for the detection of EV-A71 in stool specimens. Suspensions of stool specimens (*n* = 10) were spiked with C4-SEP06 EV-A71 (final concentration 10^1^ TCID_50_/ml) and a fixed amount of IC (10^5^ RNA copies) was added. RNA was then extracted and amplified by real-time RT-PCR. The original C4-SEP06 EV-A71 suspensions at 10^4^ and 10^1^ TCID_50_/ml in PBS were used as positive control. The stool specimens and PBS without spiking were used as negative controls. Amplification curves for EV-A71 RNA templates and the IC control from the same experiments are shown in panel **(A)** and **(B)**, respectively.

For both spiked and non-spiked stool specimens, a chloroform treatment was performed prior to RNA extraction, in accordance with WHO recommendations for Poliomyelitis or HFMD surveillance, to make them suitable for viral isolation through cell culture inoculation. To evaluate the possible effect of chloroform treatment, an EV-A71 C4-SEP06 viral suspension in PBS (10 TCID_50_/ml) was treated before RNA extraction. Untreated suspensions (10^4^ and 10 TCID_50_/ml) were also included as positive controls. Chloroform treatment showed no effect on the results of EV-A71 and IC amplification data (Figures 4A,B).

EV-A71 was consistently amplified from all contaminated stool specimens, with a mean Ct value of 32.2 ± 0.9 for nine of the 10 specimens, consistent with the positive control, i.e., EV-A71 C4-SEP06 (10 TCID_50_/ml in PBS) (Figure 4A). A shift in EV-A71 amplification was observed for one stool specimen, with a Ct value of 36.7. This shift may result from the co-extraction of PCR inhibitors from the specimen since the IC was not amplified from this specimen. The IC sequences were consistently amplified from all other non-spiked stool specimens (Figure 4B).

**FIGURE 5:**
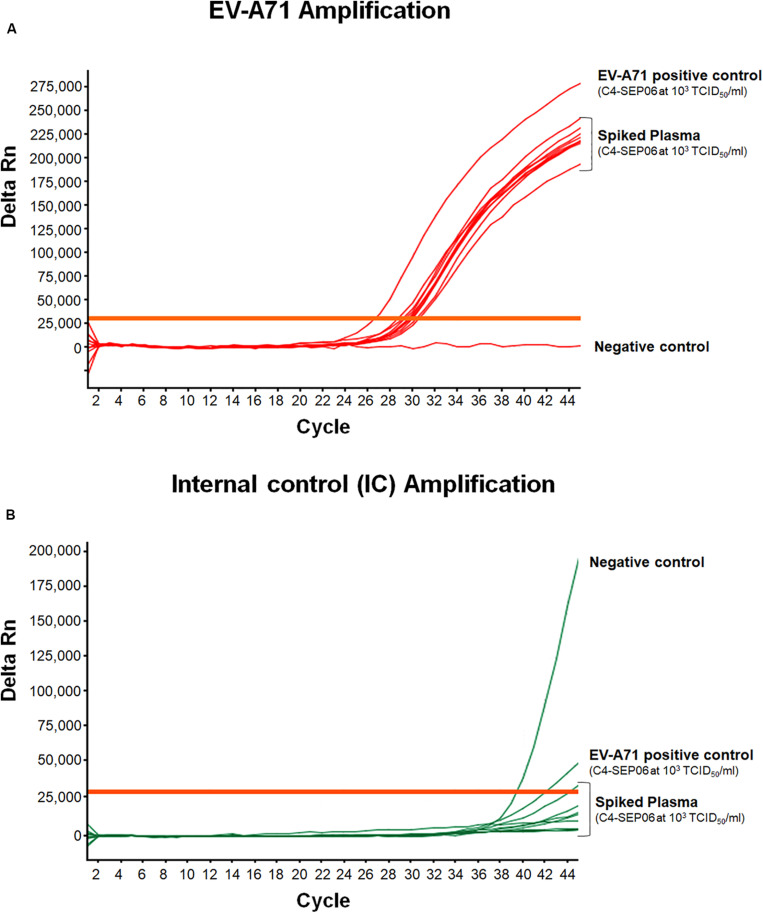
Application of the real-time RT-PCR for the detection of EV-A71 in plasma-EDTA samples. Plasma samples (*n* = 10) were spiked with fixed amounts of C4-SEP06 EV-A71 (final concentration 10^3^ TCID50/ml) and IC (10^5^ RNA copies) was added. PBS alone and C4-SEP06 (10^3^ TCID50/ml) in PBS were used as negative and positive controls, respectively. RNA was extracted and amplified by real-time RT-PCR. EV-A71 RNA curves are shown in **(A)** and the corresponding IC curves are shown in panel **(B)**.

For spiked plasma, EV-A71 amplification was observed for all specimens, with a mean Ct value of 29.8 ± 0.6 (Figure 5). These values are relatively shifted compared to those obtained from the original C4-SEP06 suspension at 10^3^TCID_50_/ml in PBS. Similarly, a shift of values was observed for IC amplification from non-spiked plasma samples (not shown). These shifts are consistent with the co-extraction of small amounts of PCR inhibitors from all plasma specimens (i.e., in the presence of heparin or EDTA).

Thus, the EV-A71 RNAs extracted from all stool and plasma specimens were directly and efficiently amplified. However, the data confirmed the utility of adding an IC to tested clinical specimens, to assess the presence of potential PCR inhibitors and therefore to check the quality of the RNA extracts used.

### Application of the Real-Time RT-PCR Assay in the Surveillance of the Circulation of EV-A71 in Africa and Madagascar

Neurological manifestations represent the most threatening manifestations of EV-A71, evoking poliomyelitis in the case of acute flaccid paralysis (AFP). Poliomyelitis surveillance implies the viral investigations of fecal samples from human cases with AFP and sewage samples collected in some critical environmental sites in accordance with WHO protocols (World Health Organization [WHO], 2004). Briefly, stool and sewage extracts are used for viral isolation through the inoculation of rhabdomyosarcoma human (RD) cells that are highly sensitive to most human enteroviruses and L20B Mouse cells expressing the human polio receptor that can be specifically infected by polioviruses. Isolates inducing cytopathogenic effect on RD cells and not L20B cells are considered to be non-polio isolates (mainly non-polio enteroviruses). These non-polio isolates constitute an interesting biological material to detect and to evaluate the circulation of other enterovirus types. The EV-A71 real-time RT-PCR test was used in a collaborative study with the Centre Pasteur du Cameroun at Yaoundé and the Institut Pasteur of Algiers, Tunis, Dakar, and Antananarivo to retrospectively analyzed non-poliovirus isolates and other biological specimens (CSF, stool extracts). A total of 1894 samples from 2000 to 2016 were tested for the presence of EV-A71 RNA (Table 3). Of the 1894 samples analyzed 20 were found positive for EV-A71 including: 17 samples from AFP (isolates or clinical specimens), one sample from an healthy patient and two samples from the environment (sewage). Regarding their biological specimen matrix: 17 were from differential cell culture non-polio isolates, and 3 samples from Tunisia were directly amplified from stool specimens of patients with AFP and without any preliminary viral isolation on cultured cells.

**TABLE 3:** Summary of the EV-A71 surveillance in Africa using the real-time RT-PCR assay.

**Country^a^**	**Period**	**Specimen tested**	**Sample origin**	**Nb. of samples**	**Nb. of EV-A71**
Algeria	2009–2015	Isolates^b^	Poliovirus survey^e^	21	0
		Isolates	AFP	117	1
		Isolates	Sewage	85	0
Tunisia	2000–2016	Isolates	AFP	137	2
	2011–2015	Stool^c^	AFP	109	3
	2004–2014	CSF	Meningitis/encephalitis	272	0
Sub Saharan	2007–2015	Isolates	Healthy	38	1
		Isolates	AFP	451	2
		Isolates	Sewage	79	2
		*Isolates*^d^	*AFP*	*5*	*5*
Madagascar	2014–2017	Isolates	Poliovirus survey	330	2
Cameroon	2009–2013	isolates	AFP	250	2
Total				1894	20

*Cerebro Spinal Fluid (CSF), Accute Flaccid Paralysis (AFP). ^*a*^Implicated Institutes: Institut Pasteur (IP) of Algiers, IP of Tunis, IP of Dakar, IP of Antanarivo, Centre Pasteur of Cameroon, respectively. ^*b*^Isolates (mainly non-polio enterovirus isolates) refer to positive cell culture isolation assays, in accordance with WHO protocols. ^*c*^Stool specimens associated with negative cell culture isolation assays. ^*d*^These isolates had been already tested positive for EV-A71 using another screening method were retrospectively tested with the new assay. ^*e*^Indicate isolates from AFP cases or healthy contacts, sewage or other origin collected through polio surveillance activities.*

Some positive samples from the Institut Pasteur of Algiers, Tunis, Antananarivo and Dakar were subjected to Sanger nucleotide sequencing of the 1D^VP1^ gene. In two positive samples high-throughput gene sequencing was done to characterize the enterovirus present in mixtures. Results of high-throughput gene sequencing indicated a mixture of EV-A71 with echovirus-20 and with echovirus-6 for the isolates of Algeria and Madagascar, respectively. Unfortunately, complete sequencing of the 1D^VP1^ gene from one of the two Madagascan EV-A71 isolates was not possible due to the very low amount of genetic material of EV-A71 compared to that of echovirus 6.

**FIGURE 6:**
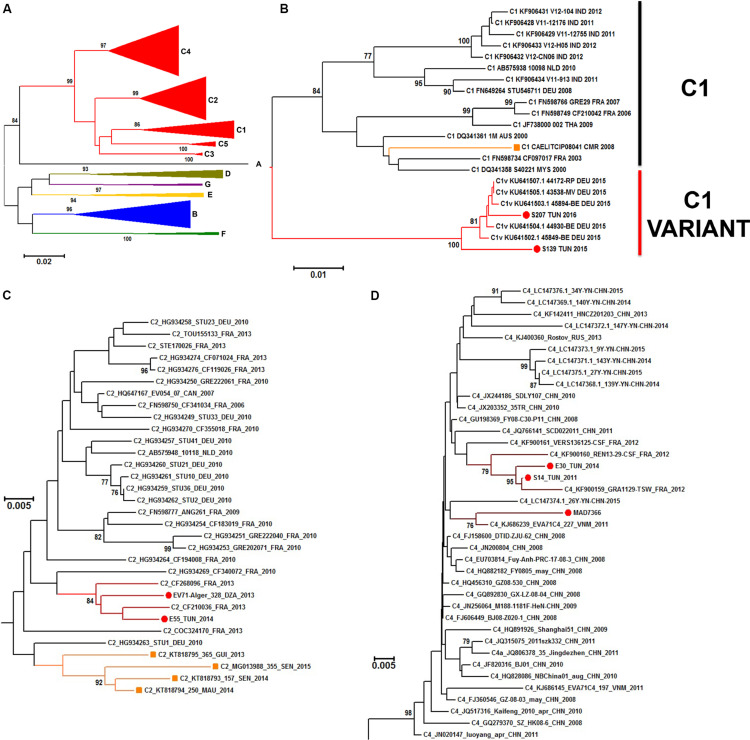
Phylogenetic relationships of some identified EV-A71 isolates. Phylogenic tree based on the complete VP1 sequence of EV-A71 retrieved from genbank database (*n* = 203 sequences), EV-A71 (sub)genogroups are denoted by colored lines **(A)**. Details of the subgenogroups C1 **(B)**, C2 **(C)**, and C4 **(D)** are shown. The recently identified EV-A71 isolates from Algeria, Tunisia, and Madagascar are indicated by a red dot. EV-A71 from the subgenogroup C1 previously reported in Cameroon and EV-A71 from the subgenogroup C2 previously reported in Senegal, Mauretania and Niger are denoted with orange branches and squares. The evolutionary distances were computed by Neighbor Joining analysis using the Kimura-2-parameter method with 1000-Bootstrap replicates to estimate node consistencies. Only percent of bootstrap >75 are indicated. The length of the branch is proportional to the number of nucleotide divergence.

A neighbor joining phylogenetic analysis was done with the new EV-A71 1D^VP1^ sequences obtained in this work. Other EV-A71 sequences available in sequences databanks were also selected to include representative of the different EV-A71 genogroups (A–G) (Figure 6A). EV-A71 isolates found in sub-Saharan countries (*n* = 5) were assigned to the genogroup E and the subgenogroup C2 (Fernandez-Garcia et al., 2018). EV-A71 isolates found in Madagascar (*n* = 1) was assigned to the subgenogroup C4. Isolates S139 and S207, originating from Tunisia and isolated in 2015 and 2016, respectively were consistently assigned to the subgenogroup C1. This subgenogroup includes European strains, and in particular, a new variant of EV-A71 genogroup C1, firstly described in Germany in 2015 (Bottcher et al., 2016) (Figure 6B). Isolates E55 and 328 from Tunisia (2014) and Algeria (2013), respectively, were consistently assigned to the subgenogroup C2. Both isolates were related to strains isolated in France during the same year but were relatively distant from strains reported in Mauretania, Senegal, and Guinea (Fernandez-Garcia et al., 2018) (Figure 6C). Isolates S14 (2011) and E30 (2014) from Tunisia were consistently assigned to the subgenogroup C4, along with isolates collected in France in 2012 (Figure 6D).

Taken together these results validate the robustness and usefulness of the real-time RT-PCR developed in this study to specifically detect EV-A71 in a broad screening analysis of numerous samples from clinical or environmental surveillance. Furthermore, phylogenetic analysis of new isolates confirms the tight link between the EV-A71 strains circulating in Europe and in North Africa.

## Discussion

EV-A71 is one of the main causes of HFMD outbreaks in Asia and Europe, and EV-A71 infection can lead to severe neurological disorders of medical concern. The circulation of this virus has been studied in detail in Asian and European populations, but not in Africa, where new genogroups (E and F) have recently been reported. The discovery of these new genogroups raises questions about their epidemiological surveillance and the emergence of new epidemic lineages with potential implications for the development of specific vaccines and treatments. In this study, we developed a novel in-house internally controlled real-time RT-PCR for the specific detection of EV-A71 in biological samples. This assay, which can be completed in 3 h, would help to improve the surveillance of EV-A71 strains belonging to the canonical genogroups B and C with a worldwide circulation, and to the recently reported, but poorly described genogroups.

The molecular detection of EVs in biological samples has become the gold standard for the diagnosis of EV infections Many different techniques have been described (Monpoeho et al., 2002; Dierssen et al., 2008; Bennett et al., 2011; Volle et al., 2012), but they target the highly conserved 5′UTR region of the EV genome. The use of this region facilitates the rapid and sensitive detection of enteroviruses in samples, but precludes identification of the type of virus. The identification of EV type requires the amplification and sequencing of the 1D^VP1^ gene encoding the VP1 capsid protein, which harbors most of the EV epitopes (Oberste et al., 1999). However, the genomic sequence of 1D^VP1^ displays considerable nucleotide variability between and within EV types (Oberste et al., 1999; McWilliam Leitch et al., 2012; Hassel et al., 2015). The development of a real-time RT-PCR specifically amplifying the 1D^VP1^ sequence of a particular EV type is challenging, because this assay must target at least three different sites for the primers and probe. Furthermore, to avoid both false-positive and false-negative results, these hybridization sites must be specific for a particular EV type, but non-specific for the various subtypes of that type. In the particular case of EV-A71, eight different genogroups have been described to date (A–H). The primers and probe used in this assay were carefully designed from the most conserved EV-A71 1D^VP1^ regions. An analysis of EV-A71 samples from various genogroups demonstrated the reliable specificity of the assay, which displayed consistent repeatability and reproducibility, below a tolerance threshold of 5% variability. The incorporation of degenerate bases into the primer and probes sequences might have made the assay less specific, but the absence of detection for various EV samples of different types demonstrated that the assay was actually highly specific for EV-A71.

The use of degenerate primers and probes in the assay resulted in some variability in the sensitivity and intensity of amplification between EV-A71 strains, regardless of the genogroups to which they belonged. However, the sensitivity values obtained for our real-time RT-PCR assay, particularly for the new EV-A71 viruses from genogroups E and F, were consistent with those reported in other studies targeting the canonical genogroups B and C (Hwang et al., 2013; Zhang et al., 2014; Thanh et al., 2015). The recently described genogroups D and G were taken into account in the design of primers and probe, and tested in collaboration with the Enterovirus Research Centre of Mumbai in India (Saxena et al., 2015). EV-A71 from genogroup D was successfully detected by the assay. Surprisingly, EV-A71 from genogroup G failed to be detected, despite its *in silico* complementarity with the used oligonucleotides. It is difficult to conclude about the efficiency of the assay to detect this genogroup since only two rare specimens were available and tested. Possibly, a weak amount of viral genetic material or a higher LOD for this genogroup may explain these negative results. Complementarity of the oligonucleotides primers with the recently described but not yet publicly available isolates of genogroup H was also confirmed (Majumdar et al., 2018).

A competitive free RNA IC was included in the assay. Its direct incorporation into the sample before RNA extraction made it possible to monitor the entire process of sample analysis (Xiao et al., 2009; Volle et al., 2012; Zhang et al., 2014). The IC was designed to co-amplified with the primers used for the specific amplification of the EV-A71 template. Unlike the hybridization sites within the EV-A71 template, which are variable, necessitating the design of degenerate oligos, those of the IC are fixed, making it possible to amplify the IC reproducibly, whatever the EV-A71 template tested. However, given its potential for interference, we paid attention to the molecular design of IC, to prevent cross-hybridization with the EV-A71 template and to ensure the optimal amplification of both templates. The inclusion of the IC in the assay had little effect on the overall sensitivity of the assay. The IC amplification began to be inhibited from concentrations of about 10^3^ TCID_50_/ml EV-A71. IC amplification thus displayed competitive sensitivity to average amounts of EV-A71, but was well amplified with 10^8^ TCID_50_/ml poliovirus in suspension.

As this real-time RT-PCR was developed for use in surveillance laboratories, we assessed its ability to detect the EV-A71 target directly in different biological specimens. RNA extracts from stool and plasma samples experimentally spiked with EV-A71 C4-SEP06 were consistently tested as positive. A detection assay on stool specimen matrix showed that this test would be easy to integrate into the routine surveillance of flaccid paralysis based on fecal specimens (World Health Organization [WHO], 2004). In particular, the chloroform-treated and clarified stool suspensions used to inoculate cell cultures for enterovirus isolation can be used directly for the specific detection of EV-A71. Throat swab specimens are considered to be useful specimens for EV-A71 detection (Ooi et al., 2010), but such specimens were not available for testing in this study. Unlike RNA extracted from stool specimens, the RNA extracted from throat swab specimens rarely contains PCR inhibitors (Buckwalter et al., 2014). It was important to show that contaminated plasma could be successfully tested with the newly developed assay because (i) a viremic stage occurs in casual infections with EVs, (ii) during neurological complications of EV-A71 infection, blood samples may be easier to obtain, and in larger quantities, than CSF samples, for which detection rates are low (less than 5%) (Ooi et al., 2010). However, plasma may be associated with more potent PCR inhibitors due to the use of anticoagulant (e.g., EDTA, which chelates the bivalent cations required for polymerase activity) than serum or CSF samples. The frequent presence of inhibitors in plasma and stool samples, as seen in some of our experiments, provided substantial support to the inclusion of the IC in the assay.

The real-time RT-PCR assay was used in a collaborative study to retrospectively analyze EV isolates from the poliovirus surveillance in Algeria, Tunisia, sub-Saharan countries, Cameroon, and Madagascar. More than 1800 samples were successfully tested in different laboratories indicating the robustness of the method. However, only 15 samples were found positive for EV-A71 on a panel of 1889 samples tested with the real-time RT-PCR assay. This low proportion of EV-A71 is consistent with previous studies of EV isolates in Cameroon and Central African Republic with ratios of 0.01 EV-A71 isolates (Bessaud et al., 2012; Sadeuh-Mba et al., 2013). Interestingly, the original isolates from Algeria and Madagascar were a mixture of different EV types, thus preventing a simple and rapid genotyping by classical sequencing methods. The use of high-throughput gene sequencing uncovered the presence of an echovirus 20 and an echovirus 6 (EV from species B) in addition of the EV-A71, thus validating the sensitivity of our EV-A71 real-time RT-PCR assay.

Phylogenetic analyses of sequences of some of the identified EV-A71 isolates classified them in the subgenogroup C1, C2, and C4, and in the genogroup E. Interestingly, EV-A71 isolates from Tunisia and Algeria displayed close molecular relationships with other strains which were shown to have circulated in Europe during the same period of time, including a newly described variant of the subgenogroup C1 associated with an outbreak of EV-A71 in Europe in 2015–2017 (Bottcher et al., 2016). This result would suggest a co-circulation of EV-A71 strains between Europe and North Africa as previously reported for other EVs between France and Tunisia (Othman et al., 2016).

In conclusion, we developed a robust and sensitive one-step real-time RT-PCR assay for the specific and sensitive detection of EV-A71 in biological specimens or cell culture supernatants, regardless of their genogroups. This reliable technique will be a useful tool for improving EV-A71 surveillance, and, in particular, for increasing our knowledge about the circulation of the new EV-A71 genogroups in Africa and other parts of the world.

## Data Availability Statement

The datasets presented in this study can be found in online repositories. The names of the repository/repositories and accession number(s) can be found below: https://www.ebi.ac.uk/ena, LR798434–LR798440.

## Ethics Statement

All human specimens were obtained in accordance with national and international ethics requirements, with the informed consent of adults healthy volunteers from the ICAReB’s Diagmicoll cohort, whose protocol was approved by the Committee of Protection of Persons Ile de France-1 (2008, April 30) and registered by the French national security agency for medicines and health products (ID-RCB: 2008-68 COL) and the US Clinical trials database (NCT03912246) (Esterre et al., 2020).

## Author Contributions

RV and FD conceived and designed the experiments and wrote the manuscript. RV, M-LJ, MF-G, RR, DR, SS-M, LB-A, and JD performed the experiments. RV, MF-G, KN, RR, J-MH, DR, SS-M, LB-A, MS, JD, M-LJ, MB, and FD analyzed the data. MF-G, KN, RR, J-MH, DR, SS-M, LB-A, MS, JD, M-LJ, and MB were involved in critical revision. All authors contributed to the manuscript and approved the submitted version.

## Conflict of Interest

The authors declare that the research was conducted in the absence of any commercial or financial relationships that could be construed as a potential conflict of interest.

## Footnotes

1 https://www.european-virus-archive.com
